# Robotics Perception and Control: Key Technologies and Applications

**DOI:** 10.3390/mi15040531

**Published:** 2024-04-15

**Authors:** Jing Luo, Xiangyu Zhou, Chao Zeng, Yiming Jiang, Wen Qi, Kui Xiang, Muye Pang, Biwei Tang

**Affiliations:** 1 School of Automation, Wuhan University of Technology, Wuhan 430070, China; jingluo@ieee.org (J.L.); xiangyuz126@126.com (X.Z.); xkarcher@126.com (K.X.); 2 Chongqing Research Institute, Wuhan University of Technology, Chongqing 401135, China; 3 Department of Informatics, University of Hamburg, 22527 Hamburg, Germany; chaozeng@ieee.org; 4 School of Robotics, Hunan University, Changsha 410082, China; ymjiang@hnu.edu.cn; 5 School of Future Technology, South China University of Technology, Guangzhou 510641, China; wenqi@scut.edu.cn

**Keywords:** robot sensors, robot control, robotic applications

## Abstract

The integration of advanced sensor technologies has significantly propelled the dynamic development of robotics, thus inaugurating a new era in automation and artificial intelligence. Given the rapid advancements in robotics technology, its core area—robot control technology—has attracted increasing attention. Notably, sensors and sensor fusion technologies, which are considered essential for enhancing robot control technologies, have been widely and successfully applied in the field of robotics. Therefore, the integration of sensors and sensor fusion techniques with robot control technologies, which enables adaptation to various tasks in new situations, is emerging as a promising approach. This review seeks to delineate how sensors and sensor fusion technologies are combined with robot control technologies. It presents nine types of sensors used in robot control, discusses representative control methods, and summarizes their applications across various domains. Finally, this survey discusses existing challenges and potential future directions.

## 1. Introduction

**Figure 1:**
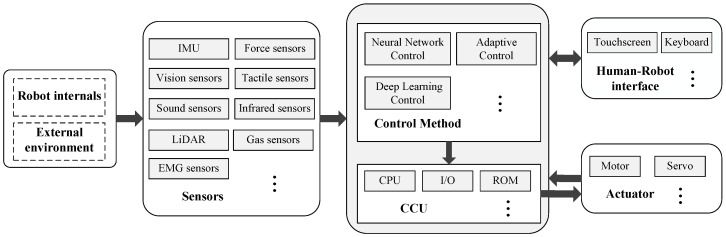
Structure of robotics system with sensors.

The advent of robots represents a significant milestone in technological evolution. Robots are now increasingly prevalent across a broad spectrum of applications, from industrial processes [1,2,3,4,5] and medical surgeries [6,7,8,9] to various real-world scenarios [10,11,12,13,14]. The advancement of robotics technology is fundamentally propelled by the development and integration of sensor technologies, which equip robots with essential tools for effective environmental interaction. In particular, advancements in sensor technology have empowered robots to perceive complex environmental conditions with greater accuracy, thereby laying the groundwork for autonomous navigation, obstacle avoidance, and task execution. Advanced sensors provide rich environmental data and, when integrated with artificial intelligence (AI) and machine learning (ML) technologies, enable robots to process this information and make informed decisions. As sensor technology continues to evolve, it enables robots to operate in increasingly variable and uncertain environments, thereby enhancing their adaptability and flexibility. Furthermore, the integration of various sensor types allows robots to achieve a more comprehensive understanding of their surroundings, thus enhancing their perception and decision-making capabilities [15]. This technology of multisensory information fusion is crucial for executing complex tasks, including high-precision manufacturing and advanced surgical assistance. The structure of robotics system with sensors is shown in Figure 1.

**Figure 2:**
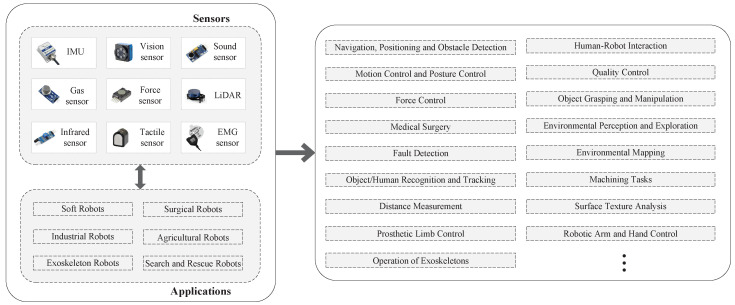
Sensors in robotic control.

This article dedicates its interest to a comprehensive overview of the role of sensors in robot control technology—dividing them into proprioceptive and exteroceptive types—and to examining their applications within robot control systems. Proprioceptive sensors, measuring internal states such as speed and joint angles, and exteroceptive sensors, gathering information from the robot’s environment, including distance and temperature, are crucial for robots to execute complex tasks with high precision and autonomy. By exploring the development and application of proprioceptive and exteroceptive sensors, this paper highlights how robots can surpass traditional limitations, thereby achieving unprecedented accuracy, adaptability, and autonomy. From enhancing manufacturing processes and surgical precision to navigating complex environments for rescue missions, sensors not only expand the capabilities of robots but also enable new applications that were previously considered impractical. This review emphasizes the symbiotic relationship between robot control technology and sensor technology, thus predicting a future where robots play a central role in addressing society’s most pressing challenges. It highlights the transformative impact of sensor technology in enhancing the capabilities and efficiency of robots. Through this exploration, the article aims to elucidate the critical role of sensors in advancing robot control technology and their potential to innovate industries by improving quality, safety, and efficiency. The relationship between robots equipped with various sensors and different application scenarios is shown in Figure 2.

The purpose of this work is to review the aspects of sensor technology in robot control technology from the perspectives of key technologies, applications, and challenges. The review is divided into four sections. The second section briefly introduces nine different types of sensors and their key technologies in robot control. The third section presents the application of robot control systems equipped with various types of sensors across different domains, such as assembly, quality inspection, minimally invasive surgical assistance, and search and rescue. Finally, the fourth section provides a conclusion and brief discussion.

## 2. Sensors and Robotics Control

Sensors are devices that are capable of perceiving and converting environmental information into electrical signals or other required formats according to specific rules, as well as transmitting them to other devices. Robots utilize a variety of sensors to detect different aspects of their environment. Generally, sensors are divided into two main categories based on their operating principles [16]. Proprioceptive sensors are used to measure the internal values of a dynamic system (such as a robot), like motor speed, robot arm joint angles, and robot pose. This article will introduce the proprioceptive sensors used in robot control applications, including Inertial Measurement Units (IMUs), magnetometers, accelerometers, and gyroscopes. Exteroceptive sensors, in contrast, acquire information from a robot’s environment, such as, measurements of distance, light intensity, sound amplitude, temperature, force magnitude, gas concentration, and image information. Therefore, the measurements obtained from exteroceptive sensors are interpreted by the robot to extract meaningful environmental features. In robot control applications, exteroceptive sensors include tactile, force, ultrasonic, infrared, LiDAR, gas, sound, vision, and EMG sensors, which will also be discussed in this article.

### 2.1. IMUs

The IMU, which is a pivotal sensor system, plays an indispensable role in the realm of robotic control. Tasked with the collection of data from robotic entities, these units transmute raw data into essential insights pertaining to localization, orientation, and acceleration. An IMU comprises an array of sensors, including but not limited to gyroscopes, accelerometers, and magnetometers. Additionally, it may encompass barometers, temperature sensors, pressure sensors, and attitude sensors. Progress in technology has ushered IMUs into an era of miniaturization, thus resulting in Microelectromechanical Systems (MEMSs) that are more compact, agile, and efficient. The utilization of IMUs in robotic control spans a diverse range of applications, from navigation and positioning to human–robot interaction and motion control.

In the field of navigation and positioning, Gao et al. [17] proposed a novel method for determining the position of indoor mobile robots by combining visual and inertial sensors. This technique utilizes an adaptive and fading extended Kalman filter to fuse data from visual sources and IMUs, thereby significantly reducing the errors commonly associated with visual navigation methods. The use of IMUs allows for frequent data updates, thereby enabling swift and accurate positioning at a lower cost than traditional laser radar solutions. However, laser radar systems exhibit superior performance in indoor localization. On the other hand, Zhao et al. [18] have developed an autonomous navigation and positioning system for serpentine robots, which is predicated on MEMS IMU technology. Operating without reliance on fixed nodes or external inputs, this solution uses the robot’s built-in MEMS IMU for navigation, thus employing an extended Kalman filter for position tracking. It stands out for its compactness, low power requirement, and straightforward installation, thereby offering a scalable option for various multilinked robotic configurations. Yet, it requires further development to support longer and more complex operations.

Regarding human–robot interaction, Chen et al. [19] presented an innovative wearable IMU sensor that employs probabilistic models to predict the initial swing phase in foot movement. Attached to the user’s right heel, the sensor accurately models foot dynamics, thus updating a probabilistic map for predicting foot placement. In addition to achieving an accuracy comparable to previous models, it enables earlier prediction times. However, it calls for improvements in creating personalized, real-time models, especially for individuals with unusual walking patterns. Škulj et al. [20] developed a system that uses wearable IMUs placed on the user’s body to remotely control collaborative robots. This approach translates the user’s motion into collaborative industrial robot commands through an innovative algorithm, which is noted for its user-friendliness, adaptability, and reliability. Future improvements aim at easing system integration and adding tactile feedback to enhance the interaction between robots and objects.

With respect to motion control, Lin et al. [21] proposed a safety control strategy for soft robots with variable stiffness based on IMU technology. By integrating IMU data with a piecewise constant curvature model, they estimated the robot’s position, orientation, and load, thereby enabling the robot to detect collisions and respond safely. These actuators offer exceptional flexibility, compliance, and versatility, all while utilizing cost-effective IMUs. However, the system faces limitations in load estimation across various positions, as well as challenges in the accuracy of position estimation, dynamics consideration, model precision, and its application in multisegmented continuum robots. Bennett et al. [9] introduced a control method for wrist rotation in myoelectric prosthetic hands using an IMU to detect upper arm movements. By detecting upper arm movements via an IMU installed on the prosthesis, this method controls the wrist’s rotational speed, thereby offering more precise control over myoelectric prostheses with wrist rotators. This control mechanism enhances task execution speed and intuitiveness for the user, thus streamlining task execution and diminishing the need for extensive task planning. Despite these advantages, the system has been discovered to be challenging, particularly when transitioning between different control regions and executing tasks that involve overhead movements.

Table 1 compares the advantages and disadvantages of different control technologies used in robots involving the IMU across various scenarios.

### 2.2. Visual Sensors

Visual sensing technology constitutes one of the quintessential means for robotic environmental perception. It functions by capturing visual data and converting it into digital formats for further utilization by robotic systems. This technology has been progressively researched and developed internationally since the 1970s. The initial prototypes of visual sensors originated in the United States. However, due to the limited computational capacity and board resources of microprocessors at that time, the supported machine vision tasks were relatively rudimentary, thus constraining its broader application. Since the 1990s, with the evolution of embedded machine vision and semiconductor technology, visual sensors have emerged as a focal point of research in both academic and industrial sectors, thereby witnessing a continuous accumulation of technological expertise. Commercially advanced products have found widespread applications in areas such as industrial manufacturing and video surveillance. Over the past few decades, researchers have explored a multitude of sensor types, including Charge-Coupled Device (CCD) image sensors [22], Complementary Metal Oxide Semiconductor (CMOS) image sensors [23], intelligent visual sensors [24,25], and infrared image sensors [26,27] to facilitate artificial vision. These sensors feature a variety of communication interfaces, including TCP/IP, OLE for Process Control (OPC), Controller Area Network (CAN), Recommended Standard 232 (RS232), etc., thereby enabling data exchange with external devices like robot controllers, Programmable Logic Controllers (PLCs), Human–Machine Interfaces (HMIs), and PCs. In robotic control, visual sensors are employed for functions such as object recognition, quality control, object grasping and manipulation, medical surgery, and autonomous navigation.

Regarding object recognition, Ji et al. [12] studied an apple-picking robot guided by an automatic visual recognition system. This system utilized a CCD camera to capture apple images, which were then processed on an industrial computer using median filters to eliminate noise. To enhance recognition precision and efficiency, a support vector machine-based apple recognition classification algorithm was introduced. While this method satisfied the recognition accuracy and efficiency requirements for apple harvesting robots, improvements are needed in recognition rates under leaf occlusion and in reducing recognition execution time in real-time systems. Li et al. [2] developed a method for the automatic skip welding trajectory recognition of spatial discontinuous welds based on laser vision sensors. They employed an adaptive angle measurement laser scanning displacement sensing system to detect the angular features of complex structures using a weld seam trajectory recognition algorithm based on Euclidean distance discrimination. This system significantly enhanced the measurement degrees of freedom—exhibiting high efficiency and stability—but its applicability needs further enhancement for wider scenarios.

With respect to quality control, Moru et al. [1] proposed a machine vision algorithm for gear quality control inspection. This algorithm acquired and analyzed images captured by a machine vision camera (Manta G-504) through a developed machine vision application that calculates relevant parameters of the gears using outer diameter, inner diameter, and tooth count algorithms. This method had extremely low system calibration errors and tolerances, thus providing high quality, but the measurement errors and precision were affected by factors such as lighting, temperature, camera resolution, and sensor configuration, which need further mitigation. Rout et al. [28] proposed a method using laser and vision sensors in robotic arc welding for detecting, locating, and setting process parameters for different weld seam gaps. They combined the seam position data obtained from vision sensors with weld seam gap changes output from laser sensors, and then they applied fuzzy logic and the NSGA-II algorithm to optimize the welding parameters, thus enhancing weld seam quality. This method achieved higher positioning accuracy and productivity, which are applicable for both continuous and offline quality control, but further refinement is needed for welds with different gaps or shapes.

In terms of object grasping and manipulation, Cao et al. [29] crafted a novel approach by integrating a multimodal neural network with Euler region regression for neuromorphic vision-based grasp estimation. Utilizing the DAVIS sensor to monitor light intensity changes at the pixel level, their network, trained on a dataset of 154 moving objects, specializes in identifying optimal grasping points. This method surpasses conventional cameras in efficiency, speed, and accuracy, thus enhancing object edge detection for improved grasp performance. Challenges include shadow misinterpretations and event density discrepancies affecting prediction reliability, thus highlighting areas for future enhancement. Wang et al. [30] proposed a method for SCARA robot pose estimation and grasping based on point cloud deep learning. They used a stereo vision system placed directly above objects to obtain point cloud data and integrated point cloud and category information into a point class vector using the end-to-end deep learning model PointNetRGPE. This method used multiple PointNet networks to estimate the robot’s grasping posture and introduced a new architecture in the PointNetRGPE model to address the issue of rotational symmetry in the z axis direction. This approach performed excellently in addressing rotational symmetry issues in z axis pose estimation, thus showing good performance, but the grasping success rate for irregular objects still requires improvement.

With respect to medical applications and surgery, Allan et al. [7] developed a method for detecting and locating surgical instruments in minimally invasive surgery. This method employed probabilistic supervised classification techniques to identify pixels belonging to surgical tools in laparoscopic images, thereby using this classification as a starting point to estimate the 3D model posture of the tools within a level set framework using an energy minimization algorithm. It was among the first methods capable of locating the five degrees of freedom posture of surgical instruments from a monocular view, without the need to rotate the instrument shaft. Nevertheless, its robustness and the accuracy of 3D estimation require improvements, and real-time performance also needs further optimization. Martell et al. [6] proposed a visual method for strain measurement in robotic surgical suturing. Through a series of steps, including image enhancement, edge detection, line detection (using Hough transform), line contour and marker detection, marker tracking (using quadratic regression), and strain calculation, they processed videos from existing surgical cameras and accurately calculated strain in the suturing thread. This method had subpixel resolution and high precision, thus providing a higher level of safety in clinical settings. However, when the suture line was at an oblique angle to the camera, the method could not accurately detect strain.

Regarding autonomous navigation, Lee et al. [31] proposed an efficient map-building method (SLAM) for indoor service robots based on a monocular vision sensor. This method directly estimated the robot’s orientation by analyzing the direction of vanishing points and derived the robot’s position and line landmark estimation model into simple linear equations. Using local map correction techniques, it effectively calibrated camera posture and landmark positions. This method reduced computational demands, thus allowing implementation in low-cost embedded systems and application in real-time autonomous robot navigation systems. Compared to other methods, it was more accurate and efficient. However, its applicability in large indoor environments needs further enhancement. Nirmal Singh et al. [32] developed a two-layered navigation system for robots blending visual and infrared sensory inputs. This system employs a hierarchical strategy, where the initial layer uses a wireless camera to capture images and define interim goals through a path optimization algorithm. Subsequently, infrared detection guides the robot towards these goals. The cycle repeats, alternating between visual mapping and infrared navigation, until the destination is reached. This method performed well under high illumination conditions, but in low light, due to the reduced computational region, the shortest path might become longer. Additionally, the system’s accuracy in outdoor environments could be impacted.

Table 2 compares the advantages and disadvantages of different control technologies used in robots involving visual sensors across various scenarios.

### 2.3. Sound Sensors

Sound sensors are widely applied and play a significant role in robotic control systems. According to their working principles, commonly used sound sensors in robotic systems include capacitive [33], piezoelectric [34], piezoresistive [35], flexible [36], and more miniaturized MEMS-type sound sensors [37]. Additionally, there exists a specialized type of sound sensor known as the ultrasonic sensor. In robotic control systems, the application of sound sensors encompasses navigation and positioning, environmental perception, and fault diagnosis. Additionally, the application of ultrasonic sensors in the field of obstacle avoidance and navigation in robotics has been introduced.

In terms of navigation and positioning, Franchi et al. [38] developed an adaptive 2D forward-looking sonar underwater navigation strategy for Autonomous Underwater Vehicles (AUVs). This approach employs a strategy based on adaptive unscented Kalman filters using 2D forward-looking sonar to estimate linear velocity. Utilizing onboard sensors, this method boasts excellent compactness, thereby making it suitable for smaller AUVs and demonstrating considerable reliability. However, its adaptability to varying environments can still be enhanced, and there is potential for further improvements in its compact design. Chen et al. [39] introduced a distributed sonar localization system for indoor robotic navigation. This system, by deploying distributed sonar transmitters on the ceiling and coordinating with sonar receivers on mobile robots, utilizes the SLAM algorithm to assist in positioning. The system is characterized by high accuracy, low cost, and easy deployment without cumulative errors, thereby offering robustness. Nonetheless, severe environmental noise interference, such as waves or reflected signals at the same frequency as the transmission signal, can adversely affect its positioning accuracy. Additionally, signal attenuation in practical scenarios limits its coverage range, thus constraining its application in large-scale indoor environments [40].

Regarding environmental perception, Uhm et al. [41] proposed a design methodology for a multimodal sensor module in an outdoor robotic monitoring system. This approach integrates multiple visual and sound sensors to form a unified system capable of synchronously extracting and matching data from 3D LiDAR sensors, thus effectively collecting information from various outdoor environments. The multimodal sensor module can gather six types of images: RGB, thermal, night vision, depth, rapid RGB, and infrared images. The system possesses good heat resistance and durability for prolonged outdoor use. Nevertheless, it requires further upgrades to withstand harsher conditions, such as polar regions, and its applicability in other settings, like medical institutions and smart factories, needs further verification. Takami et al. [42] proposed a method to estimate moving targets in an invisible field of view containing optical and acoustic sensors. Applying a recursive Bayesian estimation framework, they probabilistically processed and fused observation data from optical and acoustic sensors. This method deduces Interaural Level Differences (ILDs) from two microphones for different target positions, thereby storing these ILDs as fingerprints or acoustic clues. By comparing new acoustic observations with stored ILDs for correlation, it calculates the likelihood of acoustic observations, thus achieving accurate estimation of targets within an invisible field of view. This approach performed well across all time-steps, thus making it applicable to various practical applications like home security, health care, and urban search and rescue. However, its accuracy might be limited in complex environments.

In terms of fault detection, Yun et al. [3] developed a technique for detecting faults in robot arms using stethoscope-mounted USB microphones to capture operational sounds. This method involves training autoencoders with neural networks to distinguish anomalies from normal sounds by analyzing reconstruction errors from sound signal inputs. This method effectively reduces noise interference, thereby enhancing fault detection accuracy. However, the prediction accuracy of each stethoscope is affected by the distance between the sensor and the target. Additionally, the narrow frequency response range of the stethoscope limits its performance. Tagawa et al. [43] proposed an acoustic anomaly detection method for mechanical failures suitable for noisy real-world factory environments. Based on a noise-tolerant deep learning approach using Generative Adversarial Networks (GANs), this method reconstructs and detects anomalies in sound signals. It outperforms traditional classification methods in handling real-world industrial mechanical sound data, thereby contributing to reduced maintenance costs, enhanced safety in processing, improved equipment availability, and reduced production downtime costs while maintaining acceptable performance levels. However, this deep learning method requires extensive data when dealing with complex audio signals and industrial noise, or its performance may be compromised [44].

With respect to obstacle avoidance and navigation, Chen et al. [45] proposed a method for mobile robot navigation control using ultrasonic sensors and a Knowledge-Based Neural Fuzzy Controller (KNFC). This controller optimizes the parameters of the KNFC through a Knowledge-Based Cultural Multistrategy Differential Evolution (KCMDE) algorithm. It has been successfully applied to the PIONEER 3-DX-type mobile robot, thereby achieving efficient autonomous navigation and obstacle avoidance. The method also incorporates an innovative evasion strategy, which enhances the robot’s adaptability and navigation ability in complex environments by analyzing the angles between obstacles and the robot and setting thresholds to avoid dead zones in specific environments. Compared to other navigation methods, the KNFC demonstrates superior learning ability and system performance. However, a limitation of this approach is the need to preset multiple parameters for the differential evolution algorithm, which can pose challenges in parameter selection. Future research should focus on developing adaptive strategies for parameter adjustment, which may increase the overall complexity of the system.

Table 3 compares the advantages and disadvantages of different control technologies used in robots involving sound sensors across various scenarios.

### 2.4. Gas Sensors

Gas sensors serve as a crucial means for robots to perceive the external environment. In the field of robotic control, relevant gas sensors primarily include metal oxide semiconductor gas sensors [46], electrochemical gas sensors [47], photoionization gas sensors [48], and catalytic combustion gas sensors [49]. Gas sensors are crucial in robotic control applications, as they equip robots with the capability to detect and identify gases or volatile compounds in the environment, which is essential not only for environmental monitoring, safety assurance, and health surveillance but also provides critical information for robot–environment interaction, thereby enhancing autonomous decision making and the ability to execute complex tasks [13,50,51]. Gas sensors in robotic control systems have a broad range of applications, including navigation tracking, environmental exploration, and environmental mapping.

Regarding navigation tracking, Ishida et al. [50] innovated a method for gas/odor tracking using robots, which relies on the dynamic responses of gas sensors to detect changes in odor concentrations. This technique allows robots to adjust their speed based on sensor outputs, thus facilitating rapid and efficient odor plume tracking. This approach significantly enhances the performance of odor plume tracking robots—thereby overcoming the limitations of gas sensors—and is four times faster than existing methods. However, faster response speeds and lower power consumption might be achieved with other types of sensors. On another front, Song et al. [52] introduced a navigation system for robots combining olfactory and auditory sensors to locate odor and sound sources. This system, which employs gas and airflow sensors alongside acoustic technology for precise source localization, adapts robot movement by aligning real-time navigation with detected signals. Enhanced by wireless communication for collaborative operations, this approach offers a comprehensive environmental perception, thereby enabling complex task performance and improved adaptability. Nonetheless, outdoor navigation faces challenges from environmental variables like wind patterns and temperature fluctuations, thus highlighting areas for future enhancement.

With respect to environmental exploration and detection, Zhao et al. [14] developed MSRBOTS, a search and rescue robot system tailored for underground mines comprising two explosion-proof robots and an Operator Control Unit (OCU), all connected by a kilometer-long fiber optic cable for up to 2 km of tandem communication. Equipped with sensors, cameras, audio systems, and a unique robotic arm for obstacle removal, these robots can be operated remotely or autonomously to gather and relay environmental data. This system outperforms most other robots in water handling, obstacle clearing ability, and durability, and it has received certification from safety approval and certification centers. However, it has limited mobility and its size and weight are considerable. The designs of the robotic arm and the interface of the OCU require further optimization. Fan et al. [13] introduced a mobile robot equipped with an electronic nose for emergency gas identification and mapping. Featuring unsupervised learning for gas model updating, the robot integrates detection, discrimination, and mapping modules for comprehensive gas analysis, which are supported by radar, cameras, and scanners for enhanced awareness. It can perform online gas sensing tasks in unknown environments with strong adaptability, high efficiency, and high accuracy. In the field of robotics applications, it is imperative to ensure that sensors possess intrinsic safety, which presents a challenge for search and rescue robot systems.

In terms of environmental mapping, Loutfi et al. [53] introduced a method for environmental mapping using mobile robots equipped with gas sensors aimed at monitoring and safety applications. By incorporating spatial data from laser rangefinders, they created detailed maps showing the distribution of various odors in large indoor and outdoor settings. This approach, relying on the gas sensors’ transient responses for odor detection, facilitated the generation of multilayer maps that provide insights into gas distributions across different environments. While effective in broad settings and approximating manual survey results, the technique faces hurdles in distinguishing specific odor mixtures and requires a deeper analysis of gas sensor behaviors. Hernandez Bennetts et al. [51] developed a system for identifying and mapping multiple gases using a mobile robot fitted with a range of nonselective sensors. By leveraging an array of metal oxide sensors and probabilistic algorithms, this method effectively models gas distributions in uncontrolled environments. It uniquely applies Photoionization Detectors (PIDs) to refine gas concentration estimates and distribution models for each detected compound. This adaptive approach allows for the creation of distinct models and maps for various analytes, thereby improving prediction accuracy through PID sensor calibrations. Despite its adaptability to complex scenarios, challenges in experimental consistency, data collection, and the static nature of generated maps due to environmental variability highlight areas for ongoing research and development.

Table 4 compares the advantages and disadvantages of different control technologies used in robots involving gas sensors across various scenarios.

### 2.5. Force Sensors

Force sensors play a pivotal role in the realm of robotic control, thus boasting a wide array of applications. Over the past seven decades, significant advancements have been made in the study of multiaxis force sensors. These sensors have been extensively applied under various requirements and conditions, with prevalent sensitive element technologies encompassing resistive strain measurement [54,55], optical strain measurement [56], semiconductor strain gauges [57], and capacitive induction [58]. Depending on their structural design, multiaxis force sensors can be classified into 3-DOF force sensors [59,60], 6-DOF sensors [61], column-type force sensors [61], beam–column-type force sensors [62], and Stewart platform force sensors [63]. Within robotic control, force sensors are broadly used for force control and feedback, human–robot cooperation, object gripping and manipulation, assembly and machining tasks, and medical surgeries.

Regarding force control and feedback, Xu et al. [4] developed a hybrid force control strategy for robotic belt grinding that combines active and passive approaches to improve turbine blade manufacturing. By using a six-dimensional force/torque sensor with a PI/PD controller for active control and a one-dimensional sensor with a PID controller for passive control, this method effectively reduces grinding imperfections and inaccuracies. The integration of a Kalman filter enhances data fusion, thereby optimizing control precision and efficiency while minimizing interference. Despite its effectiveness, particularly in enhancing processing stability, the approach faces difficulties with thin-walled blades and complex shapes, thus indicating a need for further refinement in precision, system simplification, and cost reduction. Boudaoud et al. [64] introduced a model and optimal force control for a nonlinear electrostatic microgripper equipped with a force sensor for manipulating microglass balls. After assessing the microgripper’s linear range, they proposed a nonlinear model and an optimal force feedback controller using a Kalman filter to enhance signal accuracy. This method achieves precise force control, thereby ensuring reliable microobject handling with improved force resolution. However, nonlinear dynamics introduce challenges in system stability and predictability, with concerns over complexity, cost, and performance under varied conditions.

With respect to human–robot collaboration, Wang et al. [65] presented a novel approach for compensating load and calculating load information in upper limb exoskeletons using a six-axis force/torque sensor. By measuring the force and torque across the exoskeleton’s links and employing a compensator within the controller, this method allows operators to handle varying weights with consistent human–machine interaction forces. It also enables accurate determination of the load’s weight and center of gravity, which is vital for the stability of full-body exoskeletons. This method achieved effective load compensation and strength enhancement, along with precise load information computation, thereby demonstrating promising application potential. However, the complexity and cost of this system are relatively high, thus necessitating further validation in more complex and variable environments. Li et al. [66] introduced a collision detection system for robots utilizing base-mounted force/torque sensors to compute reaction forces without the need for joint friction modeling. Through a detailed procedure for dynamic model identification and a compensation technique for sensor signal coupling, this method offers improved accuracy for full-body robotic detection. This approach enhanced detection accuracy, thereby enabling full-body robotic detection suitable for advanced collision response strategies with high sensitivity and rapid detection capabilities and providing an accurate dynamic model. However, the method has not yet accurately determined collision locations, which may limit its application scope and effectiveness of response strategies, and more complex, effective collision response strategies require further development and validation.

In terms of gripping and manipulation, Ma et al. [67] introduced a robotic system designed for the precise assembly of small parts, thereby incorporating microvision and force sensing technologies. The setup includes an industrial robot equipped with a vacuum suction tool, three cameras for spatial and detailed component positioning, and a micro force sensor for insertion feedback. This system utilizes image-based visual servoing for component alignment and a force-guided approach for insertion, thus streamlining the assembly process while accommodating various component shapes. This system enhanced assembly efficiency and reduced operational complexity, thus demonstrating strong adaptability to irregular components. However, efficiency during the insertion phase of assembly remains to be improved, and the alignment accuracy needs enhancement. Sanchez et al. [68] proposed a method of blind manipulation of deformable objects based on force sensing and finite element modeling. This method senses and manipulates the deformation of soft objects using a single force–torque sensor attached to the end of a robotic arm. Instead of relying on a vision system, the method simplifies manipulation by controlling the position of individual postures on a grid, thereby achieving precise posture accomplishment. This approach possesses a certain degree of precision, but the consistency between the actual object deformation and model estimation is affected due to the difficulty in directly obtaining each object’s physical parameters. Moreover, there are certain limitations in accuracy.

Regarding assembly and machining tasks, Garcia et al. [5] introduced a dual-arm robot control device for surface treatment, thereby enabling the coordinated use of dual robot arms—one to hold the workpiece and the other equipped with a processing tool. The system allows operators to control the movement of the tool across the workpiece surface remotely, with a force sensor ensuring optimal pressure and orientation. Designed with operational constraints to prevent workspace breaches and collisions, this system marries the precision of automation with the flexibility of manual control. This system exhibits robust cooperative performance, thereby balancing the advantages of automation with user control. However, the operation is relatively complex for users, and the user interface requires further optimization to make remote operations more intuitive. Mohammad et al. [69] developed a dual-scale robotic system for polishing featuring a low-inertia force-controlled end effector for improved precision. The end effector, part of a larger microrobotic unit, utilizes a linear actuator for compliant tool movement, with an integrated force sensor providing real-time feedback for force adjustments. This setup achieved superior force tracking, thus reducing overshoot and tracking error and enhancing polishing performance. While it demonstrates flexibility and compatibility, extending its capabilities to multiaxis force control remains an area for future development.

With respect to medical surgery, Beelen et al. [70] crafted a force feedback control strategy for surgical remote manipulators to address and neutralize shunt dynamics effects, thereby enhancing operation precision and safety in medical surgeries. By compensating for parallel dynamics through a novel filter construction and employing a dual-layer method for time domain passivity, this approach significantly improves interaction fidelity with tissues, thereby offering better temporal stability and reducing injury risks. Despite its advancements in surgical precision, the technique encounters obstacles with sensor noise, bilateral stability concerns, and the capabilities of actuators and controllers, alongside a limitation in achieving comprehensive multiaxis control. Ebrahimi et al. [8] developed an adaptive control system for robot-assisted eye surgeries to safeguard against excessive scleral force. Utilizing a force-sensing device with fiber Bragg grating sensors on a robotic platform, this system employs adaptive control strategies to maintain the force within safe limits, which is guided by defined trajectories. The inclusion of a piezoelectric-driven platform to simulate surgical disturbances further underscores its utility in enhancing surgical safety and precision, most notably in retinal procedures. This approach enhances the precision and safety of retinal surgery, thus successfully reducing the force exerted on the eyeball. However, this method has training requirements for new users—implying additional time and resource investment—and further modifications and improvements are needed to enhance the acceptance and comfort of surgeons. Moreover, its application range is relatively limited, and research on users with existing robotic experience is insufficient.

Table 5 compares the advantages and disadvantages of different control technologies used in robots involving force sensors across various scenarios.

### 2.6. LiDAR

LiDAR (Light Detection and Ranging) is a technology that uses laser pulses to measure distance and speed, and it is widely utilized in robotic control and navigation systems. Depending on their operating modes, LiDAR can be classified into several main types: solid-state LiDAR [71], flash LiDAR [72], phase-shift LiDAR [73], and frequency-modulated continuous-wave (FMCW) LiDAR [74]. In robotic control, LiDAR is employed for navigation and positioning, environmental perception, target recognition and tracking, and obstacle detection and avoidance.

Regarding navigation and positioning, Li et al. [75] introduced an innovative navigation strategy for a four-wheeled legged robot by leveraging OpenStreetMap (OSM) data, 3D LiDAR, and CCD cameras to address real-world environmental variances. This hybrid approach utilizes OSM for global route planning, enhanced by Dijkstra’s algorithm, and employs sensor fusion for detailed local path adjustments, thereby ensuring accurate obstacle navigation. This method enables real-time road feature detection, thus enhancing navigation accuracy and adaptability and improving the efficiency and safety of path planning. However, the information provided by OSM may be insufficient for complex planning tasks, and future work needs to incorporate additional semantic knowledge, such as traffic signs and building shapes, to enhance the practicality of the navigation framework. Jiang et al. [10] proposed a precise autonomous navigation system for greenhouse robots combining 3D and 2D LiDAR with SLAM to streamline real-time localization and environmental mapping. The integration of various sensors facilitates a comprehensive navigation framework, thus utilizing Dijkstra for global path planning and DWA for agile local maneuvering. This method enhances the navigation system’s accuracy and environmental perception, increases safety, and reduces computational burden. The system also has a degree of scalability. However, the system’s navigation speed and accuracy are closely linked, with possible sacrifices in navigation accuracy at high speeds, and different speeds require the reconfiguration of navigation parameters, thus increasing operational complexity. Moreover, the system’s application scope is limited and may not suit more complex tasks.

With respect to environmental perception, Rovira-Más et al. [11] proposed an enhanced perception method for agricultural robot navigation specifically for navigating robots in field environments like vineyards, where GPS reliability falters. This strategy integrates 3D vision, LiDAR, and ultrasonic sensors to form an Enhanced Perception Obstacle Map (EPOM), thus improving navigation precision and obstacle avoidance. This method, integrating multiple sensing technologies, improves navigation consistency and safety while using existing sensors for self-assessment, thereby enhancing the system’s practicality and flexibility. Despite its promising capabilities, the method’s robustness across diverse conditions remains to be thoroughly validated, thus pointing toward a need for expanded testing and refinement in self-assessment techniques. Tasneem et al. [76] introduced an adaptive foveation method for scanning depth sensors, thus enabling the dynamic focusing of sensor resolution on areas of interest within its field of view. This approach, through strategic resolution allocation and deconvolution, allows for the creation of high-resolution “artificial foveae” that adapt to maximize data collection efficiency. When applied to technologies like TOF and LiDAR, it introduces possibilities for enhanced SLAM algorithm performance and energy-efficient scanning. Combined with variable angular resolution and robot motion, this method has the potential to enhance the efficiency of SLAM algorithms. However, further improvements are needed to capture and process dynamic scenes and robot motion in real-time, and more research is required to prove its advantages in capture timing and robot motion efficiency over traditional SLAM methods.

Target detection and tracking, Álvarez-Aparicio et al. [77] introduced a lidar-based method for detecting and tracking people, employing a single lidar sensor alongside the People Tracker software (PeTra, License: L-GPL v3), which incorporates a convolutional neural network (CNN) for identifying human legs in varied environments. The use of a Kalman filter enables the system to maintain consistent tracking of individuals across time. This method has shown efficacy in complex scenarios involving up to two individuals and is adaptable to platforms with limited computational power. However, its performance may be compromised in scenarios with more than two individuals. Manuel Guerrero-Higueras et al. [78] have proposed a full convolutional neural network utilizing 2D lidar scanning for tracking individuals within mobile robots, a crucial advancement for safety in cluttered environments. The PeTra tool they developed, based on an offline-trained convolutional neural network, is capable of effectively tracking legs within these complex environments. This system has potential applications in enhancing navigation, promoting human-robot interaction, and in safety-oriented applications. It utilizes lidar data to generate two-dimensional occupancy maps, which are then used in the neural network classifier. Nevertheless, the real-time performance of this system necessitates further optimization, and the current data preprocessing methodology may impact its effectiveness, requiring enhancements for improved accuracy.

In terms of obstacle detection and avoidance, Chen et al. [79] developed a LiDAR-based, real-time obstacle avoidance system that dynamically adjusts to the latest robot position and environmental changes. It uses multiconstraint functions to set subgoals within the exploration area, thereby employing an ant colony optimization algorithm for continuous path reevaluation. This strategy optimizes the use of current LiDAR data and environmental insights to efficiently plan trajectories, thus showcasing strong real-time capabilities while minimizing resource demands. Nonetheless, the approach lacks in addressing the dynamic behavior of obstacles and requires enhancements for complex settings, along with a need for better parameter adjustment methods and potential application in drone navigation for 3D space exploration. Mohd Romlay et al. [80] introduced an innovative navigation system for the visually impaired using a Fuzzy Logic Controller coupled with Optimal Reciprocal Collision Avoidance (FLC-ORCA) for maneuvering through obstacles. This system, leveraging detailed environmental data from advanced LiDAR sensors, predicts and navigates around obstacles without centralized communication, thereby relying on fuzzy logic models to account for object motion. While it significantly improves obstacle avoidance, it requires further refinement for early collision detection and should be integrated with other navigation aids for comprehensive functionality.

Table 6 compares the advantages and disadvantages of different control technologies used in robots involving LiDAR across various scenarios.

### 2.7. Infrared Sensors

Infrared sensors play a pivotal role in the field of robotic control, thus primarily utilizing infrared radiation (IR) to detect and measure the characteristics of objects. Based on their working modes, infrared sensors related to robotic control can be categorized into the following types: infrared thermal imagers [81], infrared proximity sensors [82], infrared photodetectors [83], and infrared spectroscopy sensors [84]. In robotic control, infrared sensors are used for navigation and mapping, distance measurement, human detection and tracking, posture control, and more.

Regarding navigation and localization, Xu et al. [85] have developed a novel robotic rat autonavigation system based on finite state machines. This system integrates inertial sensors and infrared thermal sensors to optimize behavior by analyzing the movements of the robotic rat and recognizing infrared targets. Sensor data are fed into a finite state machine, which is responsible for generating stimulus patterns to control the robotic rat. This system offers an innovative solution in the Search and Rescue (SAR) domain, thus leveraging the advantages of biomimicry and mechanical control. However, its design and implementation are complex, and it is currently primarily applied in specific SAR environments, with further research required to enhance its reliability and practicality. Viejo et al. [86] developed a 3D SLAM technique for robots that integrates visual cues with Growing Neural Gas (GNG) networks using data from infrared and Kinect cameras. This method enhances robot self-localization by applying GNG networks to 3D spatial data and refining movement tracking through advanced 3D registration techniques. By effectively merging 3D spatial information with 2D visual features, it achieves significant improvements in mapping accuracy and data processing efficiency. However, the computational time of the GNG algorithm is lengthy and needs further optimization for speed improvement.

With respect to distance measurement, Pierlot et al. [87] devised the Beacon-Based Angle Measurement Sensor (BeAMS), which is a novel localization tool for mobile robots using modulated infrared signals for precise angle measurement and beacon identification. BeAMS stands out for its real-time tracking capabilities, compactness, and energy efficiency, thereby achieving a high data acquisition rate suitable for dynamic applications. Its central estimator enhances the accuracy of beacon angle calculations, thus contributing to the system’s reliability. However, the automatic gain control mechanism introduces errors, and localization accuracy is susceptible to varying environmental conditions [88]. Mesa et al. [89] developed a distance estimation approach that harnesses the power of MultiLayer Perceptrons (MLPs) combined with a trio of reflective optical distance sensors—visible light, ultraviolet, and near infrared. This sensor fusion model is designed to extend the measurement capabilities and ensure redundancy, thereby improving accuracy and reducing susceptibility to interference by compensating for different radiation effects. The MLP framework allows for customization with multiple configurations, thus enhancing the system’s adaptability for embedded applications. Despite its benefits, the necessity for extensive MLP pretraining could pose challenges in terms of time and resource allocation.

In terms of human detection and tracking, Liu et al. [90] developed a mobile robot-assisted system for detecting elderly falls using Pyroelectric Infrared (PIR) sensors for body contour imaging and posture recognition. This innovative approach uses PIR sensors not just for motion detection but to create detailed contour images for posture analysis, thus employing sparse representation techniques for fall detection. This cost-effective method is unaffected by lighting conditions but relies on frontal capture, with data collection possibly limited by participants’ postures. Additionally, environmental heat sources may interfere with the infrared imaging, thus performing better in more controlled settings like nursing homes. Benli et al. [91] proposed a Thermal Multisensor Fusion (TMF) method aiming to achieve human-centric tracking through thermal vision and human thermal signals. This method combines omnidirectional infrared sensors and stereoscopic infrared sensors, where the former provides a broad field of view for detecting human targets, and the latter determines the distance of the human body in specific directions. By fusing data from these two sensors, the system can more accurately predict target distance. The method achieves high-precision tracking through TMF stereoscopic distance results on multiple platforms, thus effectively improving localization accuracy. It enables tracking independent of lighting conditions and can track targets in a wider field of view. However, this method is currently limited to tracking a single target and requires increasing the number of cooperative robots equipped with TMF and introducing other types of sensors to enhance accuracy.

Regarding posture control, Chou et al. [92] introduced a biomimetic stair-climbing method based on a hexapod robot. Utilizing a two-phase process involving the initial body tilt for front leg positioning and subsequent center of mass adjustment, this method integrates infrared rangefinders and inclinometers for stair detection and body tilt measurement, respectively. This approach ensures the robot’s effective adaptation to various stair dimensions, thereby enhancing its autonomous climbing ability. This method endows the robot with strong autonomous climbing capabilities and good environmental adaptability, but its data collection may be limited by the viewing angles, and its design and implementation are relatively complex. Additionally, the robot’s autonomous movement in broader environments requires improvement. Li et al. [93] proposed a method for the real-time detection of gait events in lower limb exoskeleton robots using infrared distance sensors. By using smart shoes integrating three infrared distance sensors, stable distance signals are obtained and converted into effective foot posture information. This system uses the gap between the heel and toe for real-time online detection of six gait events throughout the gait cycle. The system employs an online detection algorithm using a local search window and fixed threshold for minimal time delay and lower computational load. This method enhances the accuracy, detection rate, and response speed of gait event detection and can be effectively integrated with exoskeleton robot systems. However, this system is currently mainly applicable to regular gait patterns, with further research and optimization needed for more complex gait modes.

Table 7 compares the advantages and disadvantages of different control technologies used in robots involving infrared sensors across various scenarios.

### 2.8. Tactile Sensors

Tactile sensors, as a key technology for robots to perceive their external environment, have received widespread attention in the last decade. These sensors, by measuring the interaction between the robot and its environment, emulate biological tactile perception. The primary aim of tactile sensing technology is to detect or perceive physical quantities during robot–object or robot–environment interactions to gather information about objects and environments or to complete specific operational tasks. Tactile sensors are pivotal in robotic control applications, as they endow robots with human-like tactile capabilities, thereby enabling them to perceive and interact with their operational environment more precisely and enhancing their capacity to execute complex tasks with increased safety and efficiency [94,95,96,97]. Since the 1970s, research on robotic tactile sensors has progressed alongside the evolution of robotic technology—undergoing nearly fifty years of development—which can be segmented into three phases: the 1970s, the 1980s to the 1990s, and from 2000 to the present.

Over the past several decades, researchers have explored a multitude of approaches to create artificial tactile sensations [98,99,100,101]. These include various types of flexible and stretchable sensors such as resistive [102], piezoresistive [103,104], capacitive [105], optical [106], piezoelectric [107,108], and acoustic sensors [109]. Whether used independently or in combination, these sensors have made significant contributions to simulating human tactile perception, though there still exists a considerable gap compared to the tactile perception capabilities of humans. Some key developments in tactile sensing or electronic skin (e-skin) in robotics have introduced new possibilities in the field of robot control. These include surface texture analysis, force control and feedback, object recognition and classification, and gripping and manipulation control.

Regarding surface texture analysis, Wang et al. [94] crafted a novel tactile sensor array capable of distinguishing surface textures and grooves during sliding actions by employing Finite Element Modeling (FEM) and phase delay algorithms for in-depth analysis. This 3 × 3 multilayer sensor array effectively captures variations in normal forces and utilizes phase delay algorithms to discern different textures and inclinations. This method enhances the precision of sliding detection and surface texture recognition, and the structural design of the sensor array is relatively complex, thus requiring further research and algorithmic optimization for complex surface textures. In a subsequent study, Wang et al. [110] explored the use of a wearable tactile sensor array for advanced surface texture recognition, thereby employing a combination of the WMB model and artificial neural networks. The WMB model, integrating the W-M function with beam bundle theory, aids in reconstructing quasi-three-dimensional surface profiles and simulating force fluctuations during sliding. By analyzing Characteristic Frequency Clusters (CFCs) and employing neural networks for data classification, this method achieves high classification accuracy but is complex in model and algorithm, thus making it primarily suitable for specific types of surface textures. Future research should explore the mechanical responses of tactile sensor arrays in robotic hand contact, compression, and sliding motions.

With respect to force control and feedback, Deng et al. [95] proposed a method to stabilize objects by controlling the gripping force of a multifingered robotic hand through tactile sensing, thereby enabling it to stabilize objects with precise grip force adjustments based on tactile feedback. Utilizing a deep neural network to process tactile data for material and contact event recognition and a Gaussian mixture model for force and location estimation, the system dynamically modulates the gripping force. The system effectively integrates multifunctionality and efficient tactile detection capabilities for precise force control. However, its limitation lies in only sensing local contact information between the robotic hand and the object, thereby necessitating integration with other sensing technologies (like vision or force/torque sensors) to enhance perceptual capabilities. Armleder et al. [111] introduced an innovative force control system for human–robot interaction, thereby employing a large-scale robotic skin for full-body tactile feedback. This system facilitates complex physical collaborations by providing sensitive touch feedback and enabling multipoint contact management and adaptive interaction with humans and objects. Through the integration of tactile and proximity data, the robot can perform a variety of tasks with enhanced safety and efficiency. Despite its promising application in interactive scenarios, the system’s adaptability to diverse interaction types remains to be fully explored, thus indicating a need for further refinement to extend its utility across a wider range of human–robot collaboration contexts.

In terms of object recognition and classification, Pohtongkam et al. [96] unveiled a tactile recognition system for humanoid robots featuring a sophisticated sensor array and Deep Convolutional Neural Networks (DCNNs) for object classification. Their palm-sized sensor array, crafted using PCB technology and conductive materials, facilitates detailed pressure distribution mapping. By evaluating 19 different DCNN architectures for identifying objects across 20 categories, they demonstrated the potential for enhanced tactile-based object recognition. Despite its high accuracy, the system demands considerable computational efforts and extensive training, thereby highlighting the need for efficient image processing and machine learning strategies to optimize performance. Pastor et al. [112] introduced an innovative approach for tactile object recognition through robotic palpation, thereby employing 3D convolutional neural networks to process pressure image sequences as 3D tactile tensors. This technique, which is capable of discerning both external and internal object characteristics under varying grasp forces, leverages tactile sensor arrays for detailed pressure imaging. Named 3D TactNet, their CNN model effectively identifies diverse and complex object types, including deformable items. The system recognizes a variety of object types, including complex and deformable objects, thus demonstrating high recognition performance and adaptability. However, its design and training process are relatively complex, and it may misclassify very similar categories. Future research needs to explore multimodal perception strategies and new dynamic methods.

Regarding grasping and manipulation control, Stachowsky et al. [97] developed a novel sliding detection and correction approach to optimize robotic gripping force, thus making it adaptable to a variety of grippers and not reliant on prior knowledge of the object’s properties. This method employs a sliding signal detector to assess the extent of slip and a force setpoint generator to adjust the grip strength accordingly. Aimed at preventing slippage while avoiding unnecessary force, it proves particularly beneficial for handling fragile items. The technique has shown broad applicability and effectiveness in preventing slip without excessive force across multiple tests. Nonetheless, its effectiveness is contingent on certain conditions, thereby indicating a need for further enhancements to ensure stability across all manipulation tasks. Calandra et al. [113] introduced an innovative robotic grasping technique that leverages both visual and tactile inputs to iteratively refine gripping actions. By developing an action-conditional model that learns from initial sensory data, their system predicts the success of different regrasping strategies, thus allowing for the iterative selection of optimal actions. This deep learning approach, trained on thousands of trials, simplifies the development of grasping strategies by circumventing the complexities of tactile sensor calibration and contact force modeling. While the method successfully merges visual and tactile data for improved grasp performance, its current limitation to single-step predictions and its lack of fine manipulation capabilities before gripping or in response to slippage during lifting points to areas for future development. The goal is to apply this model in more challenging environments to broaden its application scope.

Table 8 compares the advantages and disadvantages of different control technologies used in robots involving tactile sensors across various scenarios.

### 2.9. Electromyography (EMG) Sensors

Electromyography (EMG) sensors are devices designed to capture the electrical signals generated by muscles during their activity [114,115]. Based on their operational principles and application modes, they can be categorized into four primary types: surface EMG sensors (sEMG) [116,117], intramuscular EMG sensors (iEMG) [118], multichannel EMG sensors [119], and wireless EMG sensors [120]. Within the realm of robotic control, EMG sensors are applied in the control of prosthetic limbs, robotic arms and hands, and the operation of exoskeletons.

Regarding prosthetic limb control, Tavakoli et al. [121] have developed an innovative control strategy for advanced prosthetic hands utilizing single-channel sEMG signals to differentiate up to four discrete gestures. These gestures, encompassing fist clenching, hand opening, wrist flexion and wrist extension, and facilitating the actuation of the prosthetic limb’s grasping functions. Employing high-dimensional feature spaces coupled with support vector machine algorithms, this methodology enables the efficacious classification of said gestures. Distinguished by its simplicity, rapidity, and cost-effectiveness, this system is noted for its compactness, lower energy consumption, and enhanced user intuitiveness. Nonetheless, it may pose operational challenges to users under specific scenarios. Furthermore, Cha et al. [122] have proposed a novel methodology that integrates EMG signal classification with rule-based tactile feedback mechanisms for the control of robotic prosthetic hands. This method employs convolutional neural networks (CNNs) for the classification of EMG signals procured from subjects, thereby facilitating intent recognition. Additionally, a wearable tactile feedback device has been conceptualized to provide users with grasp force information pertaining to the robotic prosthetic. This integrated system, integrating CNN models with tactile feedback devices, assures efficacious control over the prosthetic hand while delivering intuitive feedback. Despite its notable precision and accuracy, the considerable dimensions of the tactile feedback apparatus present operational difficulties, and the prerequisite for extensive EMG signal classification training introduces supplementary temporal costs.

With respect to robotic arm and hand control, Bouteraa et al. [123] introduced a technique for the remote manipulation of robotic arms that integrates biofeedback, thereby employing gestures captured by Kinect sensors and EMG signals for controlling robotic hands while incorporating force feedback to enhance grasping actions. This system utilizes visual and electromyographic signals for posture recognition and modulates grasping intensity through a fuzzy logic system based on EMG signals, thereby achieving precise trajectory tracking. This methodology amalgamates multiple sensing technologies, thus ensuring high efficiency, stability, and robustness of operation. Future endeavors will focus on optimizing control strategies to enhance system adaptability and user convenience. Laksono et al. [124] have devised an upper limb robotic control scheme based on EMG signals, which maps human elbow and shoulder movements to a two-degree-of-freedom robotic arm, to facilitate human–robot collaboration and remote operation. By connecting three EMG sensors to the brachioradialis, biceps brachii, and anterior deltoid muscles, this framework captures muscle activity signals to control the robotic arm’s motion. This architecture offers an effective method for the direct control of robots through human muscle signals that is characterized by simplicity, low computational cost, rapid response, and strong robustness. Future work will concentrate on enhancing control strategies through pattern recognition technologies to improve system performance and adaptability. Zeng et al. [125,126] proposed to use EMG signals to estimate a human user’s arm stiffness and then developed a Dynamical Movement Primitives (DMPs)-based method that simultaneously models the human user’s movement and stiffness, thus enabling the transfer of compliant manipulation skills from the human user to robots.

In terms of the operation of exoskeletons, Gui et al. [127] proposed an EMG-driven torque estimation method for custom lower limb exoskeletons, thus enabling the adaptive prediction of two-degree-of-freedom joint torques. Utilizing Radial Basis Function Neural Networks (RBFNNs) and an enhanced Slotine–Li controller, this approach eliminates the need for the calibration of traditional EMG torque models. The introduction of a dual learning mechanism allows the system to adapt in real-time to variations in EMG signals, thereby improving operational flexibility and accuracy. This adaptive strategy simplifies the usage process and avoids frequent recalibration, thus demonstrating its potential and convenience for practical applications. However, the system currently faces limitations in addressing unknown Ground Reaction Forces (GRFs). Caulcrick et al. [128] have developed a torque modeling technique for lower limb exoskeletons that integrates Mechanomyography (MMG) and EMG signals, thereby aiming for more accurate on-demand assistive control. Employing machine learning techniques such as linear regression, polynomial regression, and neural networks for human joint torque prediction, they explored the complementary and competitive advantages of MMG and EMG signals in exoskeleton interaction. This method not only enhances torque estimation accuracy and stability but also reveals potential applications in the rehabilitation of neuromotor disorders, with future research directed towards expanding sensor networks to optimize system performance.

Table 9 compares the advantages and disadvantages of different control technologies used in robots involving EMG sensors across various scenarios, and Table 10 compares the advantages and disadvantages of various sensor types in robotic control applications.

## 3. Applications

Over the past few decades, as sensor technology has increasingly integrated with robotics, robot control techniques utilizing various sensors have been widely applied in different fields. This section will introduce several typical applications of robot control technologies using different sensors in fields such as quality inspection, minimally invasive surgical assistance, and search and rescue. As demonstrated in Figure 2, we will not elaborate further here.

### 3.1. Industrial Field

With the development of electronics, computer science, mechanical engineering, and artificial intelligence, robots equipped with various sensors are playing an increasingly indispensable role in the industrial sector. The advancement of Industry 4.0 has led to stringent requirements for precision and accuracy in intelligent factories, especially in gear measurement and inspection. In this context, high-quality control has become central to gear manufacturing inspection. To meet this need, Moru et al. [1] developed a machine vision algorithm that achieves subpixel level accurate measurement of gears through image analysis.

In welding tasks, such as robot welding for complex box beam structures, Li et al. [2] addressed the issue of low efficiency and flexibility in teaching programming by proposing a weld seam trajectory recognition method based on laser scanning displacement sensors. This method allows for the automatic guidance of the welding gun in spatial intermittent jumping welding. In [28], a robot arc welding technique assisted by laser and vision sensors was presented, which improves weld position accuracy and welding quality by adjusting the welding gap value within the welding cycle.

Sound and vibration analysis are key tools for diagnosing the health of machines. Yun et al. [3] introduced a method using an internal sound sensor based on a stethoscope for anomaly detection in industrial robot arms using an autoencoder. In [43], an acoustic anomaly detection method for mechanical failures in noisy real-world factory environments was developed.

In grinding and polishing tasks, Xu et al. [4] proposed a hybrid active–passive force control strategy for robot sand belt grinding of turbine blades aimed at reducing grinding marks and improving contour accuracy. In [5], a dual robotic arm control method for surface treatment tasks was presented. Mohammad et al. [69] developed a low-inertia effect force-controlled end effector for robotic polishing. In the field of precision operations, Ma et al. [67] developed an automated precision robotic assembly system equipped with microvision and force sensors. In [64], a method for modeling the optimal force control of a nonlinear electrostatic microgripper was proposed.

### 3.2. Medical Field

Minimally invasive surgery is favored for its small incisions, short hospital stays, and rapid postoperative recovery. Surgical robots in this field have enhanced the visualization and precision of operations, thus further expanding the range of minimally invasive surgeries. One challenge in robotic surgery is maintaining appropriate suture tension, and to address this, Martell et al. [6] developed a method for assessing surgical suture strain using visual measurement. This method, based on noninvasive video processing, can display the strain of the suture line in real-time, thereby effectively reducing the learning curve and enhancing the performance and safety of robotic surgery. In advanced robot-assisted and computer-assisted surgery, research on detecting and locating surgical instruments in laparoscopic images is a crucial component. Allan et al. [7] proposed a detection and localization method for instruments in laparoscopic surgery.

With respect to ophthalmic surgery tasks, Ebrahimi et al. [8] introduced an adaptive control strategy to enhance the safety of scleral force in robot-assisted ophthalmic surgery, thereby aiming to reduce the risk of unsafe scleral forces. In the field of prosthetic control, ref. [9] developed a method for controlling the rotation of a myoelectric prosthetic wrist based on an IMU, which is aimed at improving the control of myoelectric prostheses with wrist rotators.

### 3.3. Agricultural Field

The development of intelligent greenhouses is closely linked to the application of mobile robots. In the complex environment of greenhouses, the precise positioning and navigation of robots are key technologies. Jiang et al. [10] proposed an autonomous navigation system for greenhouse mobile robots combining 3D LiDAR and 2D LiDAR SLAM. To address the autonomous navigation of agricultural robots operating in orchards, ref. [11] developed a technology combining 3D vision, LiDAR, and ultrasonic sensors for enhanced perception navigation in agricultural robots.

In the field of apple-picking robots, a key technology is the machine vision system for identifying and locating apples. Ji et al. [12] developed an automatic recognition method guided by a vision system for apple picking robots.

### 3.4. Rescue and Search

Emergency personnel such as firefighters, bomb technicians, and urban search and rescue experts often face extreme dangers in natural and man-made disasters, including hazardous chemicals in the air. Mobile robots equipped with gas sensors can provide crucial information, such as identifying and locating potential sources of different chemicals in emergency areas. Fan et al. [13] proposed a method for gas identification and mapping in emergency response scenarios using mobile robots equipped with electronic noses. Zhao et al. [14] presented a remote sensing rescue robot system for coal mine underground environments consisting of an operation control unit and two mobile robots with explosion-proof and waterproof capabilities capable of remotely observing and collecting information about the coal mine environment.

## 4. Conclusions and Future Directions

In this review, we briefly introduced nine types of sensors applied in robot control technology, including IMUs, visual sensors, acoustic sensors, gas sensors, force sensors, LiDAR, infrared sensors, tactile sensors, and EMG sensors. We discussed their specific applications in robot control and categorized and elucidated representative control methods. Furthermore, we provided an overview of their applications across various domains. Lastly, we explored potential future research directions.

In the realm of sensor technology and robotic control, our investigation has spanned two pivotal sensor types: proprioceptive and exteroceptive, each with distinct applications in various robotic scenarios. Proprioceptive sensors, including IMUs, gyroscopes, accelerometers, and magnetometers, equip robots with detailed insights into their own status and movement dynamics. These sensors are indispensable across a spectrum of functionalities such as navigation, positioning, interactive human–robot dynamics, and precise motion regulation. Conversely, exteroceptive sensors—encompassing tactile, force, ultrasonic, infrared sensors, LiDAR, gas sensors, acoustic sensors, visual sensors, and EMG sensors—empower robots to sense and engage with their external environment effectively. Technological advancements have significantly accelerated the evolution of these sensors, thus rendering them more compact, efficient, and economically viable. Moreover, improvements in algorithmic and computational methodologies have substantially enhanced our capacity to process and interpret the vast amounts of data that these sensors generate. This convergence of technological progress has unlocked new avenues for robot autonomy, adaptability, and intelligence, thereby broadening their applicability across diverse fields. Robots are now increasingly capable of performing in a wide array of roles, ranging from industrial automation and medical procedures to personal assistance and emergency response tasks, thus heralding a new era in robotics where machines can operate more seamlessly and intelligently within human environments.

The vigorous development of robot control technology is contingent on the rapid advancement of sensor technology and other related technologies. However, future research and applications in this field are expected to encounter multifaceted challenges and new directions of work. As specific tasks become increasingly complex and refined, future research will necessitate the development of new sensor technologies, thereby continuously enhancing the sensitivity and accuracy of sensors to meet the demands for precise detection and identification in more complex environments. A robot’s ability to perceive its environment is fundamental to performing complex tasks. Future efforts will require more in-depth research into how to effectively integrate and fuse information from diverse sensors (such as visual, tactile, sound, and gas sensors) to enhance robots’ understanding of their environment and the accuracy of their decision making. The challenge lies in efficiently processing and interpreting a large volume of multimodal data and ensuring the effective integration of information among different sensors.

## Figures and Tables

**Table 1:** In the field of robotics control technologies, comparisons of the advantages and disadvantages of different methods involving the IMU across various scenarios.

Application Scenario	Technology/Method Name	Advantages	Disadvantages/Improvements Needed
Navigation and Positioning	Indoor Mobile Robot Positioning Method [17]	High data update frequency, rapid and accurate positioning, and low cost.	Performance is inferior to LiDAR in indoor positioning.
	Serpentine Robot Autonomous Navigation and Positioning System [18]	Small size, low energy consumption, easy installation, and low computational demand.	Needs improvement for long-duration and long-distance applications.
Human-Robot Interaction	Probabilistic Distribution Model-Based Method for Predicting Foot Placement in Early Swing Phase [19]	Can predict earlier while maintaining similar accuracy.	Needs improvement in adapting to users with abnormal gaits.
	Flexible Remote Operation Method for Collaborative Industrial Robots [20]	Intuitive and easy to use, high flexibility, and strong robustness.	System needs further integration simplification and the addition of tactile feedback loops.
Motion Control	Active Safety Control of Variable Stiffness Soft Robots [21]	Excellent flexibility, compliance, multifunctionality, and low cost.	Unable to perform load estimation at any position, with limitations in dynamics consideration and model accuracy.
	Radial Forearm Myoelectric Prosthesis Wrist Rotation Control Method [9]	Makes task execution quicker, more intuitive for the user, and reduces the need for task planning.	Difficult to operate, especially when switching controls and performing overhead tasks.

**Table 2:** In the field of robotics control technologies, comparisons of the advantages and disadvantages of different methods involving visual sensors across various scenarios.

Application Scenario	Technology/Method Name	Advantages	Disadvantages/Improvements Needed
Object Recognition	Apple-Picking Robot Guided Automatic Recognition Visual System [12]	Improved recognition precision and efficiency.	Lower recognition rate under leaf occlusion, and real-time performance needs improvement.
	Automatic Welding Trajectory Recognition Method for Spatial Intermittent Weld Seams [2]	High degree of measurement freedom and efficiency, strong stability.	Applicability needs further enhancement.
Quality Control	Machine Vision Algorithm for Gear Quality Control Inspection [1]	Extremely low system calibration error and tolerance, high quality.	Measurement error and precision are affected by lighting and other factors.
	Method for Detecting, Searching, and Setting Process Parameters for Different Weld Seams Gaps in Robotic Arc Welding [28]	Higher positioning accuracy and productivity; suitable for continuous and offline quality control.	Needs further improvement for welds with different gaps or shapes.
Object Grasping	Multimodal Neural Network Estimation of Grasping Posture Based on Euler Region Regression [29]	More energy-efficient, lower latency, higher temporal resolution, and dynamic range.	Shadows of objects may be mistakenly processed, affecting prediction results. Accurate prediction is difficult for objects with insufficient events.
	SCARA Robot Pose Estimation Grasping Method Based on Point Cloud Deep Learning [30]	Good performance in dealing with the pose estimation problem of z axis rotational symmetry.	Lower success rate in grasping irregular objects.
Medical Surgery	Method for Detecting and Locating Surgical Instruments in Minimally Invasive Surgery [7]	Capable of locating the five degrees of freedom of surgical instruments, thus reducing the positioning process.	Robustness and accuracy of 3D estimation can be improved, and real-time performance needs further optimization.
	Strain Measurement Method in Robotic Surgical Suturing [6]	Subpixel resolution, high precision, and high safety.	Unable to accurately detect strain when the suture line is at an angle to the camera.
Autonomous Navigation	Efficient Map-Building (SLAM) Method for Indoor Service Robots [31]	Low computational requirements, low cost, applicable to embedded systems, high real-time performance, accurate and efficient.	Applicability in large indoor environments needs further improvement.
	Dual-Level Subgoal Mobile Robot Navigation Algorithm [32]	Performs well under high illumination conditions.	In low light conditions, the shortest path may become longer. In outdoor environments, accuracy may decrease.

**Table 3:** In the field of robotics control technologies, comparisons of the advantages and disadvantages of different methods involving sound sensors across various scenarios.

Application Scenario	Technology/Method Name	Advantages	Disadvantages/Improvements Needed
Navigation and Positioning	Adaptive 2D Forward-Looking Sonar Navigation Strategy [38]	Excellent compactness and reliability.	Adaptive capability to variable environments could be improved.
	Split-Type Sonar Localization System [39]	High precision, low cost, easy deployment, no cumulative error, strong robustness.	Severe noise in the environment can easily affect positioning accuracy; has limited coverage.
	Knowledge-Based Neural Fuzzy Controller (KNFC) Method [45]	Superior learning capability and system performance compared to other navigation methods.	Requires presetting multiple parameters for differential evolution algorithms, thus posing challenges in parameter selection.
Environmental Perception	Design Method for Multimodal Sensor Module [41]	Good heat resistance and durability for long-term outdoor use.	System needs further upgrades to adapt to harsher conditions.
	Estimating Invisible Moving Targets [42]	Performs well across all time-steps; suitable for a variety of practical applications.	Accuracy may be limited in complex environments.
Fault Detection	Industrial Robotic Arm Anomaly Detection [3]	Effectively reduces noise interference, thereby improving fault identification accuracy.	Stethoscope’s prediction accuracy is affected by distance, and its narrow frequency response range limits performance.
	Acoustic Anomaly Detection in Noisy Industrial Environments [43]	Outperforms traditional classification methods, thus reducing maintenance and production downtime costs, as well as enhancing safety and equipment availability.	Requires large amounts of data; otherwise, performance may be compromised.

**Table 4:** In the field of robotics control technologies, comparisons of the advantages and disadvantages of different methods involving gas sensors across various scenarios.

Application Scenario	Technology/Method Name	Advantages	Disadvantages/Improvements Needed
Navigation Tracking	Control Method for Gas/Odor Plume Tracking Robots [50]	Significantly improved tracking performance, thus overcoming the limitations of gas sensors.	Using other types of sensors could achieve faster response times and lower power consumption.
	Mobile Robot Navigation Method for Odor/Sound Source Searching [52]	Enhanced perception and reaction to the environment. Capable of performing more complex tasks, with stronger collaboration and information sharing capabilities.	Natural disturbances may impact navigation performance.
Environmental Exploration	Remote Sensing Search and Rescue Robot System (MSRBOTS) Designed for Coal Mine Environments [14]	Strong water-crossing ability, obstacle clearance capability, and durability.	Limited maneuverability, large size and weight, the design of the robotic arm and the interface of the operation control unit need further optimization.
	Method for Gas Detection and Mapping in Emergency Response Scenarios by Mobile Robots [13]	Strong adaptability, high efficiency, and accuracy.	Ensuring intrinsic safety of sensors poses a challenge.
Environmental Mapping	Method for Mapping Gas Distribution of Multiple Odor Sources [53]	Suitable for large, unmodified environments, thus achieving effects similar to manual operations.	Untrained systems face challenges in classifying specific mixtures of odors.
	Method of Combining Multiple Nonselective Gas Sensors on Mobile Robots [51]	Improved prediction accuracy through the incorporation of calibration factors of PID sensors.	Data collection process is challenging, the accuracy of gas distribution needs improvement, and the distribution maps are static.

**Table 5:** In the field of robotics control technologies, comparisons of the advantages and disadvantages of different methods involving force sensors across various scenarios.

Application Scenario	Technology/Method Name	Advantages	Disadvantages/Improvements Needed
Force Control	Mixed Active/Passive Force Control Strategy for Robotic Belt Grinding [4]	High precision and efficiency, strong adaptability, improved processing stability.	Lower machining precision for complex parts, complexity and cost need reduction.
	Modeling and Optimal Force Control Method for Nonlinear Electrostatic Microgrippers [64]	Enhanced gripping force resolution, higher reliability, accuracy, practicality, and applicability.	Stability and predictability may be affected by nonlinear behavior, complexity, and cost need reduction.
Human–Robot Collaboration	Multijoint Load Compensation and Load Information Calculation for Upper Limb Exoskeletons [65]	Effective load compensation and strength enhancement.	High complexity and cost.
	Robotic Collision Detection Method [66]	Improved detection accuracy, high sensitivity, and rapid detection capability; accurate dynamic model provided.	Inability to precisely determine collision location.
Object Manipulation	Efficient Assembly Method for Small Components by Automatic Precision Robots [67]	Increased assembly efficiency, reduced operational complexity, strong adaptability to irregular parts.	Lower efficiency in assembly insertion phase; alignment accuracy needs improvement.
	Blind Manipulation Method for Deformable Objects [68]	Certain precision in the method.	Consistency and accuracy limitations between actual object deformation and model estimation.
Machining Tasks	Dual-Arm Robot Control Device for Surface Treatment Tasks [5]	Powerful cooperative performance; balanced advantages of automation and user control.	Relatively high operational difficulty; user interface needs further optimization.
	Low-Inertia Effect Force-Controlled End Effector for Robot Polishing [69]	Reduced overshoot, stable time, and tracking error, excellent force tracking ability, high flexibility, and compatibility.	Further research needed for multiaxis force control implementation.
Medical Surgery	Force Feedback Control Design for Nonideal Teleoperators [70]	Improved precision, reduced risk of accidental damage, enhanced temporal stability of interaction.	Affected by force sensor noise, bilateral stability, and limitations of actuators and controllers; incomplete implementation of multiaxis control.
	Adaptive Control Method to Enhance Scleral Force Safety in Robot-Assisted Ophthalmic Surgery [8]	Improved precision and safety in retinal surgery; successfully reduced force on the eyeball.	Requires additional time and resources; further improvements needed to increase acceptance and comfort for surgeons.

**Table 6:** In the field of robotics control technologies, comparisons of the advantages and disadvantages of different methods involving LiDAR across various scenarios.

Application Scenario	Technology/Method Name	Advantages	Disadvantages/Improvements Needed
Navigation and Positioning	Autonomous Navigation Method for Quadrupedal Robots [75]	Real-time detection enhances navigation accuracy and adaptability, improves path planning efficiency and safety, and has good robustness.	May not be sufficient for complex planning tasks; needs increased practicality.
	Autonomous Navigation System for Greenhouse Mobile Robots [10]	Improves navigation precision and environmental perception, enhances safety and reduces computational load, has certain scalability.	Navigation precision may be sacrificed at high speeds, and navigation parameters need to be reset for different speeds.
Environmental Perception	Enhanced Perception Method for Agricultural Robot Navigation [11]	Combines multiple sensing technologies to improve navigation consistency and safety; enhances system practicality and flexibility.	Real-world application effectiveness needs to be validated.
	Adaptive Foveation Method for Scanning Depth Sensors [76]	Offers more flexible data collection and has the potential to improve SLAM algorithm efficiency.	Real-time performance needs improvement; advantages in capture time and robot motion efficiency to be verified.
Target Tracking	Method for Person Detection and Tracking Using LiDAR Sensors [77]	Performs well in complex scenarios with no more than two people; has a lower computational burden.	May perform poorly in scenarios with more than two people.
	Method for Tracking People in Mobile Robots Based on Fully Convolutional Networks [78]	Has certain applicability.	Real-time performance needs further optimization; data preprocessing methods need improvement to enhance accuracy.
Obstacle Detection	Real-Time Multiconstraint Avoidance Strategy Based on LiDAR [79]	Good real-time performance, reduces computational and storage costs, lowers complexity.	Dynamic characteristics of obstacles not fully considered; needs better methods to fine-tune cost function parameters.
	Robot Navigation Assistance Device Using Fuzzy Logic Controllers [80]	Significantly improves obstacle avoidance capability.	Limited as a standalone application; needs integration with other navigation systems for best performance.

**Table 7:** In the field of robotics control technologies, comparisons of the advantages and disadvantages of different methods involving infrared sensors across various scenarios.

Application Scenario	Technology/Method Name	Advantages	Disadvantages/Improvements Needed
Navigation and Localization	Novel Autonomous Navigation System for Robot Mouse Based on Finite State Machines [85]	Fully utilizes the advantages of biomimicry and mechanical control.	Design and implementation are relatively complex; reliability and practicality need to be improved.
	Method Combining Visual Features and Growing Neural Gas Network for Robot 3D SLAM [86]	Effectively reduces camera errors, reduces data amount, and improves parallel processing. Enhances the accuracy and efficiency of feature extraction.	GNG algorithm’s computation time is long; needs further optimization for speed enhancement.
Distance Measurement	Beacon-Based Angle Measurement Sensor (BeAMS) for Mobile Robot Localization [87]	High acquisition frequency (10Hz), strong real-time capability, compact, low power consumption, flexible and easy to use, and high accuracy.	Localization accuracy may be affected by environmental diversity changes.
	Distance Estimation Method Combining Multilayer Perceptron (MLP) and Reflective Optical Sensors [89]	Low cost, high accuracy and anti-interference ability, flexible architecture choice, suitable for embedded systems.	The system requires pretraining of MLP, which may consume additional time and resources.
Human Tacking	Mobile Robot-Assisted Contour Imaging and Body Posture Recognition Method for Elderly Fall Detection [90]	Cost-effective; unaffected by lighting conditions.	Relies on frontal capture, and environmental heat sources may interfere with infrared imaging.
	Human Tracking with Thermal Multisensor Fusion (TMF) Method [91]	High accuracy, achieves tracking independent of lighting conditions, and can track targets in a broader field of view.	Limited to tracking a single target; accuracy has room for improvement.
Posture Control	Bionic Stair Climbing Method Based on Hexapod Robots [92]	Endows robots with strong autonomous climbing capabilities and good environmental adaptability.	Data collection may be limited by viewing angles, and design and implementation are relatively complex.
	Method for Real-Time Detection of Gait Events in Lower Limb Exoskeleton Robots [93]	Improves the accuracy, detection rate, and response speed of gait event detection.	Further research and optimization are needed for more complex gait patterns.

**Table 8:** In the field of robotics control technologies, comparisons of the advantages and disadvantages of different methods involving tactile sensors across various scenarios.

Application Scenario	Technology/Method Name	Advantages	Disadvantages/Improvements Needed
Surface Texture Analysis	Tactile Sensor Array for Sliding and Groove Surface Recognition in Sliding Motion [94]	Improved precision in sliding detection and surface texture recognition.	Structure design is relatively complex, and recognition of complex surface textures still requires algorithm optimization.
	Method for Recognizing Surface Textures [110]	Achieved high classification accuracy.	High complexity and currently mainly applicable to specific types of surface textures.
Force Control	Method for Controlling the Grasping Force of Multifinger Grippers to Stabilize Objects [95]	Effectively integrates multifunctionality and efficient tactile detection, thus achieving precise force control.	Can only perceive local contact information between the gripper and objects; needs to be combined with other sensing technologies to enhance perception.
	Human–Robot Interaction Force Control System Based on Multimodal Robot Skin [111]	Demonstrated applicability in real-world applications.	Limited to handling a finite type of user interactions; the system needs further optimization for broader application scenarios.
Object Recognition	Tactile Recognition System for Humanoid Robots Based on Deep Convolutional Neural Networks (DCNNs) [96]	Improved object recognition accuracy and efficiency.	The system has high complexity, thus requiring substantial computational resources and training time.
	Robotic Palpation Tactile Object Recognition Method Based on 3D Convolutional Neural Networks [112]	Showed high recognition performance and adaptability.	The design and training process is relatively complex, and misclassification may occur in very similar categories.
Grasping Task	Slip Detection and Correction Strategy for Adjusting Robot Gripping Force [97]	Applicable to a wide range of gripping scenarios, strong universality, and effectively eliminates slippage without excessive force.	May not be suitable in some special cases; further research is needed to expand its stability during operations.
	Robot Grasping and Regrasping Technique Integrating Vision and Tactile Information [113]	Enhanced adaptability and efficiency during the grasping process, thus showing a high success rate on various objects.	Can only perform single-step predictions, and its actions are relatively coarse, making it possibly unsuitable for precise object manipulation or slip handling before grasping.

**Table 9:** In the field of robotics control technologies, comparisons of the advantages and disadvantages of different methods involving EMG sensors across various scenarios.

Application Scenario	Technology/Method Name	Advantages	Disadvantages/Improvements Needed
Prosthetic Limb Control	Prosthetic Hand Control Technology Capable of Recognizing Four Gestures [121]	Simple, fast, low-cost, more compact system, lower energy consumption, and more intuitive user operation.	Users may find it difficult to operate in certain scenarios.
	Robotic Prosthetic Hand Control Integrating EMG and Tactile Feedback Devices [122]	High recognition precision and accuracy.	The tactile device is large and inconvenient to operate; additionally, extensive training is required for users, thus increasing additional time costs.
Robotic Arm and Hand Control	Remote Manipulation Technology for Robotic Arms with Integrated Biofeedback [123]	High efficiency, stability, and robustness.	The adaptability of the system and the convenience of use for the operator need to be improved.
	Upper Limb Robotic Control Scheme Based on EMG Signals [124]	Simple operation, low computational cost, rapid response, strong robustness.	The performance and adaptability of the system need further improvement.
Operation of Exoskeletons	EMG Signal-Driven Torque Estimation Method for Lower Limb Exoskeletons [127]	High flexibility and accuracy; possesses strong applicability.	There are limitations in dealing with unknown Ground Reaction Forces (GRFs).
	Torque Modeling Technique for Lower Limb Exoskeletons Combining MMG and EMG Signals [128]	Strong accuracy and high stability.	Awaiting optimization of system performance through joint use with other sensors.

**Table 10:** Comparisons of the advantages and disadvantages of various sensor types in robotic control applications.

Sensor Name	Advantages	Disadvantages
IMUs [9,17,18,19,20,21]	Low cost, small size, high flexibility, easy installation, and low computational requirements. Unaffected by environmental factors, with strong robustness.	Limited accuracy, not suitable for long-duration and long-distance scenarios.
Visual Sensors [1,2,6,7,12,28,29,30,31,32]	High precision, strong stability, low latency, and more energy-efficient.	Affected by lighting, obstructions, and shadows.
Sound Sensors [3,38,39,41,42,43,45]	High precision, low cost, easy deployment, excellent compactness, and reliability.	Susceptible to noise in the environment, limited coverage, and performance affected by distance.
Gas Sensors [13,14,50,51,52,53]	High efficiency and accuracy; strong adaptability.	Easily affected by environmental factors.
Force Sensors [4,5,8,64,65,66,67,68,69,70]	High precision and efficiency, strong adaptability, flexibility, and compatibility.	Complex implementation and higher cost.
LiDAR [10,11,75,76,77,78,79,80]	Strong real-time performance, high flexibility, and good robustness.	Difficult to achieve both high precision and speed in navigation applications; suboptimal performance when used alone.
Infrared Sensors [85,86,87,89,90,91,92,93]	Strong real-time performance, compact, low power consumption, low cost, flexible use, high accuracy, and unaffected by lighting conditions.	Limited by the angle of view, relies on frontal capture, and environmental heat sources may interfere with infrared imaging.
Tactile Sensors [94,95,96,97,110,111,112,113]	High recognition performance and adaptability, strong universality, high accuracy, and efficiency.	Complex implementation and needs to be combined with other sensing technologies.
EMG Sensors [121,122,123,124,127,128]	Highly accurate and precise, with notable flexibility and cost-effectiveness, alongside swift responsiveness and robust stability.	Adaptability is somewhat limited, and the operation is comparatively complex.

## Data Availability

No data were generated or used during the study.
